# Are Perceived and Objective Distances to Fresh Food and Physical Activity Resources Associated with Cardiometabolic Risk?

**DOI:** 10.3390/ijerph15020224

**Published:** 2018-01-29

**Authors:** Katherine L. Baldock, Catherine Paquet, Natasha J. Howard, Neil T. Coffee, Anne W. Taylor, Mark Daniel

**Affiliations:** 1 Centre for Population Health Research, Sansom Institute for Health Research, School of Health Sciences, University of South Australia, Adelaide SA 5001, Australia; catherine.paquet@unisa.edu.au; 2 Wardliparingga Aboriginal Research Unit, Sansom Institute for Health Research, University of South Australia, Adelaide SA 5001, Australia; natasha.howard@unisa.edu.au; 3 Centre for Research & Action in Public Health, Health Research Institute, University of Canberra, Canberra ACT 2601, Australia; neil.coffee@canberra.edu.au (N.T.C.); mark.daniel@canberra.edu.au (M.D.); 4 Population Research and Outcome Studies, Discipline of Medicine, The University of Adelaide, Adelaide SA 5001, Australia; anne.taylor@adelaide.edu.au; 5 Department of Medicine, St Vincent’s Hospital, The University of Melbourne, Melbourne VIC 3065, Australia; 6 South Australian Health and Medical Research Institute, Adelaide SA 5001, Australia

**Keywords:** perceptions, geographic information system, neighbourhood, food environment, physical activity environment, cardiometabolic risk factors, Australia

## Abstract

Perceived and objective measures of neighbourhood features have shown limited correspondence. Few studies have examined whether discordance between objective measures and individual perceptions of neighbourhood environments relates to individual health. Individuals with mismatched perceptions may benefit from initiatives to improve understandings of resource availability. This study utilised data from *n* = 1491 adult participants in a biomedical cohort to evaluate cross-sectional associations between measures of access (perceived, objective, and perceived-objective mismatch) to fruit and vegetable retailers (FVR) and public open space (POS), and clinically-measured metabolic syndrome and its component risk factors: central obesity, dyslipidaemia, hypertension and pre-diabetes/diabetes. Access measures included perceived distances from home to the nearest FVR and POS, corresponding objectively-assessed road network distances, and the discordance between perceived and objective distances (overestimated (i.e., mismatched) distances versus matched perceived-objective distances). Individual and neighbourhood measures were spatially joined using a geographic information system. Associations were evaluated using multilevel logistic regression, accounting for individual and area-level covariates. Hypertension was positively associated with perceived distances to FVR (odds ratio (OR) = 1.14, 95% confidence interval (CI) = 1.02, 1.28) and POS (OR = 1.19, 95% CI = 1.05, 1.34), after accounting for covariates and objective distances. Hypertension was positively associated with overestimating distances to FVR (OR = 1.36, 95% CI = 1.02, 1.80). Overestimating distances to POS was positively associated with both hypertension (OR = 1.42, 95% CI = 1.11, 1.83) and dyslipidaemia (OR = 1.25, 95% CI = 1.00, 1.57). Results provide new evidence for specific associations between perceived and overestimated distances from home to nearby resources and cardiometabolic risk factors.

## 1. Introduction

The clustering of cardiometabolic risk factors known as the metabolic syndrome is a global public health issue [1,2]. Population-level cardiometabolic health benefits can be achieved through engaging in behaviours protective of cardiometabolic health, such as eating well and undertaking physical activity [3]. Improving access to health-promoting resources such as public open spaces (POS) and fruit and vegetable retailers (FVR) within neighbourhoods can support residents to take up and maintain these positive health behaviours. Access to POS such as parks and recreational facilities within neighbourhoods has been positively associated with greater physical activity levels [4,5,6,7]. Similarly, local access to FVR such as supermarkets and greengrocers has also been positively associated with greater fruit and vegetable consumption [8,9,10]. Inconsistent associations have been reported, however, between FVR, POS and cardiometabolic outcomes beyond health behaviours.

Objectively determined access to FVR has been inversely associated with obesity in several studies [11,12,13,14,15], and objectively assessed POS size and greenness have been inversely associated with cardiometabolic risk [16]. Other studies utilising objective measures of environment, however, have reported null associations between obesity and access to parks [17] and supermarkets [18,19], and between fruit and vegetable store density and cardiovascular mortality [20]. Perceived local availability of physical activity resources has been inversely associated with being both obese and physically inactive [21]. Area-aggregated positive perceptions of the physical activity and healthy food environment, expressed as a composite score, have been associated with a lower incidence of type 2 diabetes [22]. Auchincloss and colleagues [23] found that positive perceptions of the physical activity environment, but not the healthy food environment, were associated with lower insulin resistance, and in a separate study reported that positive perceptions of the healthy food environment, but not the physical activity environment, were associated with lower obesity incidence [24]. A recent study including both perceived and objective neighbourhood measures reported associations between type 2 diabetes and survey-based (perceived) measures of neighbourhood healthy food and physical activity resources, but not corresponding geographic information system (GIS) objective measures [25]. 

Synthesising findings from the above studies is challenging due the diversity of measures and methods applied to represent FVR and POS, an issue highlighted by previous reviews [26,27]. Yet, each type of measure (i.e., objective and perceived) has limitations. Objective measures of neighbourhood features, such as those obtained from commercial or government databases, tend to be used more often in place-health research [11,12,13,14,15,17,18,19]. This is because they arguably provide a “concrete and absolute” account of the neighbourhood environment ([28], p. 346), are generally regularly updated and easily accessible [29], less time- and labour-intensive than direct field observations, and not subject to same-source bias. However, objective data may be subject to more error compared to direct observations or field validations [29,30,31,32,33,34,35], and may also be subject to error related to the process of geocoding [30]. Such error can bias associations between environmental measures and health behaviours and outcomes [36]. Fewer studies have used perceived measures [21,22,23,24], which not only reflect the objective reality, but also individual, neighbourhood, and societal factors [37,38,39]. 

It is undoubtedly important to know whether the actual (i.e., objectively measured) accessibility of neighbourhood resources is related to health behaviours and outcomes; however, resources that are not *perceived* to be accessible are less likely to be utilised [40,41]. Thus, the impact of the neighbourhood built environment on health is dependent upon residents’ perceptions of their neighbourhood [42]. Several authors have advocated the inclusion of both types of measure in studies evaluating relationships between neighbourhoods and health [38,43,44,45,46,47]; yet, studies to do so in relation to cardiometabolic risk are few [21,25]. 

In addition to gaining clarity on how perceived and objective neighbourhood attributes are independently associated with cardiometabolic risk, it is also necessary to determine whether the discordance between resident perceptions and objective assessments of neighbourhood features may be related to health outcomes [48,49]. Previous research has reported that, among individuals living within one kilometre of a supermarket, those who did not perceive a supermarket as within walking distance from home (i.e., perception and objective assessment mismatched) consumed less fruit and vegetables than those who perceived a supermarket within walking distance (i.e., perception and objective assessment matched) [50]. Further, in an Australian prospective study [51], it was reported that, among those living in a high-walkable area, those who perceived it to be low-walkable (i.e., mismatched perception and objective assessment) were less physically active and had higher body mass index values over a four-year period, compared to those with matched perceptions of living in a high-walkable neighbourhood. Outcome measures and perceived exposure measures in these two studies were self-reported, however, raising the possibility of same-source bias. It is unknown whether the discordance between perceived and objective access to neighbourhood resources and health outcomes extends to clinical measures of cardiometabolic risk. Knowledge of an association between cardiometabolic risk and discordance between perceived and objective food and physical activity environments for residents of well-serviced areas would contribute to the development of interventions targeting improved perceptions without necessarily requiring changes to the environment.

The present study drew on data from urban-dwelling Australian adults involved in a biomedical cohort study to evaluate associations between perceived and objective access to FVR and POS and clinically measured cardiometabolic risk. This study further sought to evaluate whether the discordance between perceived and objective distances to FVR and POS, specifically the overestimation of distances, related to cardiometabolic risk. A secondary aim was to assess whether fruit and vegetable intake, and physical activity, mediated any associations between cardiometabolic risk and FVR, and POS, respectively.

## 2. Materials and Methods

### 2.1. Study Context

**Figure 1:**
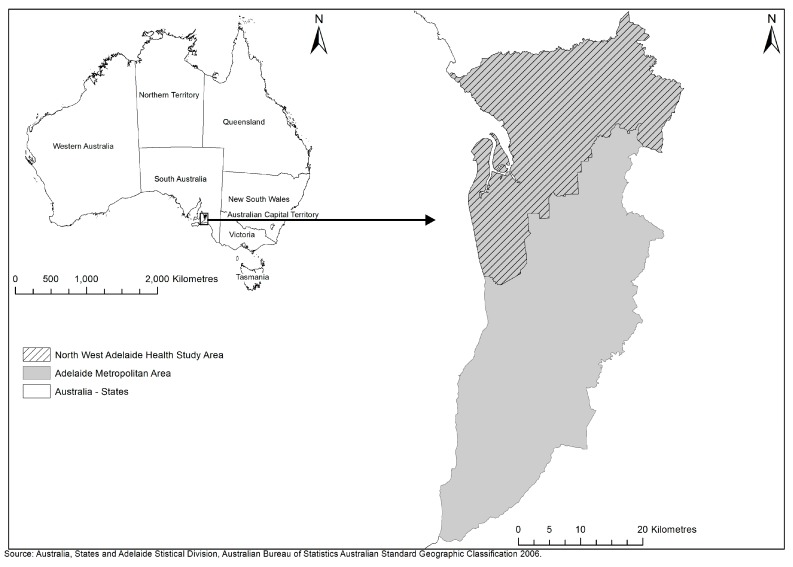
Study area (North-West Adelaide) within metropolitan Adelaide (Adelaide Statistical Division), South Australia, Australia.

This study was part of the Place and Metabolic Syndrome (PAMS) project, drawing on individual-level data from the North West Adelaide Health Study (NWAHS) conducted in the north-western region of Adelaide (Figure 1), the capital city of South Australia, Australia. Adelaide in 2006 had a population of approximately 1.1 million persons, residing within a geographic area extending 30 kilometres (km) east-west, and 80 km north-south [52]. PAMS received approvals from the Ethics of Human Research Committees of the Central Northern Adelaide Health Service (Application no.: 2010010) University of South Australia (Protocol no.: P029/10), and South Australian Department of Health (Protocol no.: 354/03/2013).

### 2.2. Sample

The NWAHS is a longitudinal cohort study with a baseline sample of 4056 randomly selected adults aged 18 years and over, and three waves of data collection to date. Participants were originally recruited between 2000 and 2003 from the northern and western metropolitan regions of Adelaide [53,54], and the second wave of data collection occurred between 2004 and 2007. NWAHS data collected across Wave 2 were utilised for this cross-sectional analysis, as this was the only period for which all required measures were available. At Wave 2, approximately 6% of NWAHS participants still residing in Adelaide had moved outside the north-west region. Self-reported responses to questions about socio-demographics, health conditions and health behaviours were obtained from a telephone interview. Biomedical measurements were obtained in a clinic by trained staff. Information on medications prescribed for participants, current at the time of the Wave 2 clinic visit, was obtained by linking Australian Pharmaceutical Benefits Scheme data to each individual participant using their Medicare number. Perceptions of neighbourhood characteristics and residential location were obtained in 2007 using a telephone follow-up survey conducted after the clinic visits. All data from participants with a valid residential address were geo-coded to enable individual data to be linked with built-environment data according to participants’ areas of residence. 

### 2.3. Measures

#### 2.3.1. Outcome Variable

The metabolic syndrome and its component measures, namely central obesity, dyslipidaemia, hypertension, and prediabetes/diabetes were used to represent cardiometabolic risk. The metabolic syndrome is a useful measure for estimating population-level risk for cardiometabolic diseases [55]. Metabolic syndrome component measures were also analysed individually, given the results of other PAMS analyses indicating specific prospective associations between built-environment attributes and individual measures of cardiometabolic risk [16,56]. 

Metabolic syndrome was classified according to International Diabetes Federation criteria [57]. These include central obesity (waist circumference ≥94 cm for Europid men, ≥90 cm for non-Europid men, and ≥80 cm for all women), plus any two of the following four factors: raised triglyceride level (>1.7 mmol/L), low high-density lipoprotein (HDL) cholesterol (<1.03 mmol/L in men and <1.29 mmol/L in women), or treatment for lipid abnormality (low HDL cholesterol or elevated triglycerides, or lipid-lowering treatment collectively classified as ‘dyslipidaemia’); raised blood pressure (systolic blood pressure ≥130 or diastolic blood pressure ≥85 mmHg), or treatment for hypertension; raised fasting plasma glucose (FPG; ≥5.6 mmol/L), or previously diagnosed type 2 diabetes. Criteria for dyslipidaemia or hypertension were considered met if a participant had been prescribed medication to treat such conditions in the six months prior to their clinic attendance. The four component measures used in analyses, including dyslipidaemia, prediabetes/diabetes, central obesity and hypertension, were each classified using the definitions given above.

#### 2.3.2. Independent Variables

##### Resident Perceptions of Fruit and Vegetable Retailers and Public Open Space

Variables indicating NWAHS resident perceptions of the walking distances to FVR and POS were derived from five questions in the land-use mix diversity subscale of the Australian version of the Neighbourhood Environment Walkability Scale (NEWS-AU) [58], a modified version of the NEWS [59]. Perceived walking distances in minutes from home to the nearest supermarket, greengrocer, park, nature reserve and sports field were reported by participants in five categories (“1–5 min”, “6–10 min”, “11–20 min”, “21–30 min”, and “more than 30 min”). Perceived distance categories were coded 1 through 5 for analysis. Supermarkets and greengrocers were collectively defined as FVR. Parks, nature reserves and sports fields were collectively defined as POS. Parks and nature reserves were classified as passive POS, whereas sports fields were defined as active POS. 

##### Objectively Assessed Fruit and Vegetable Retailers and Public Open Space

Spatial information for FVR was obtained from the 2007 South Australian Retail Database [60], and POS spatial data were obtained from the 2007 South Australian Property Cadastre provided by the Land Services Group, Department of Planning, Transport and Infrastructure, South Australian Government. Supermarkets and greengrocers were classified as FVR, corresponding to the perceived measures. POS was defined as either (i) publicly owned land parcels larger than an average residential house block (700 m squared (m^2^)) [61], with or without provisions for organised sport or physical activity; or (ii) as a publicly accessible outdoor sporting facility such as a tennis court, and classified as active (e.g., football fields or parks including tennis courts or other sporting facilities), or passive (e.g., reserves). A single POS could include multiple land parcels; therefore, to avoid over-counting, parcels within a five-metre adjacency were merged to create a single land parcel. 

The distances along the road network from the geo-coded participant residential address to the nearest FVR and to the nearest POS were measured using Arc GIS 9.3 (Environmental Systems Research Institute, Redlands, CA, USA). Distances were expressed as walking time in minutes to correspond to the perceived measures based on a moderate adult walking speed of 4.8 km (3.0 miles) per hour [62], and categorised as follows: (1) 0–400 m (1–5 min walk); (2) 401–800 m (6–10 min walk); (3) 801–1600 m (11–20 min walk); (4) 1601–2400 m (21–30 min walk); and (5) greater than 2400 m (greater than a 30 min walk). Objective distance categories were coded 1 through 5 for analysis.

##### Discordance between Perceived and Objective Distances (Overestimation of Distances) to Fruit and Vegetable Retailers and Public Open Space

Overestimation of the actual distance to FVR and POS represented discordance between perceived and objective distances. The difference between perceived and objective distance scores was calculated as objective less perceived distance. Positive difference scores indicate underestimated distances to destinations, whereas negative difference scores indicate overestimated distances (i.e., respondents perceived the distance to destinations to be further than objective distance). From the difference score, a dichotomous variable indicating whether respondents overestimated or correctly estimated distances to destinations was calculated for both FVR and POS. Those who underestimated distances to FVR or POS were excluded from analyses.

#### 2.3.3. Mediators

Fruit and vegetable intake and physical activity were included as potential mediators of associations between cardiometabolic risk and FVR and POS measures. Fruit and vegetable intake was self-reported by participants from two questionnaire items, expressed as the total number of servings of fruit and vegetables usually consumed each day. These survey items have been used previously in the Australian National Health Survey [63], and equivalent questions have demonstrated acceptable test-retest reliability (intraclass correlation coefficient (ICC) = 0.67 for fruit, ICC = 0.65 for vegetable, and ICC = 0.70 for fruit and vegetable intake) in previous research [64]. 

Physical activity was assessed using several questions also derived from the Australian National Health Survey [63], where respondents were asked to report on the frequency and duration of their walking, moderate activity and vigorous activity undertaken either for fitness, recreation or sport over the previous two weeks. The total minutes of physical activity derived from these questions has demonstrated acceptable test-retest reliability (ICC = 0.57 (95% confidence interval (CI) 0.49 to 0.68)) [65]. From these questions, a total physical activity score was calculated by multiplying the number of times the activity was undertaken in the last two weeks (number of sessions) by the average time per session by the intensity of activity, where intensity, or metabolic equivalent of task (MET), was defined for each of the three categories of exercise identified in the survey, as follows: 3.5 for walking; 5.0 for moderate exercise; and 7.5 for vigorous exercise [63]. 

#### 2.3.4. Covariates

Participant age, gender, educational attainment assessed as less than bachelor’s degree or bachelor’s degree or higher, annual household income (AUD) assessed as $20,000 or less, $20,001 to $60,000, or greater than $60,000, and the duration of residence were entered as covariates in all models estimating the associations between perceived, objective, and overestimated distances to neighbourhood resources and cardiometabolic risk. 

Area-level median weekly household income, extracted at the State Suburb level from the 2006 Australian Bureau of Statistics Census of Population and Housing [66] and ascribed to each participant based on their residential address, was included in all models to account for potential confounding by area socioeconomic status [67]. State Suburbs are a derived Census Geographic Unit which are formed by aggregating the finest Census unit to approximate the well-characterised Australian urban localities of the ‘suburb’ [68].

### 2.4. Statistical Analysis

All analyses were conducted in SAS (version 9.3; SAS Institute Inc., Cary, NC, USA). All regression models were analysed using the SAS glimmix procedure, accounting for participant age, gender, educational attainment, household income, duration at current residence, and area-level income. Statistical significance was set at alpha = 0.05.

Associations between cardiometabolic risk measures and the perceived, objective and overestimated distances to FVR and POS were estimated using multilevel logistic regression models. A random intercept was specified to account for the spatial clustering of participants within State Suburbs. Individuals were modelled at the first level and State Suburbs at the second level. A first set of regression models estimated the unique associations between perceived, objective and overestimated distances to FVR and POS and the cardiometabolic risk measures (Model 1). Perceived and objective distance variables were then entered simultaneously into a second set of models (Model 2) to estimate their independent associations with cardiometabolic risk. A third set of models (Model 3) estimated associations between environmental and cardiometabolic risk measures accounting for behaviour as part of the mediation analyses described below. 

Mediation by fruit and vegetable intake and physical activity was formally assessed for all statistically significant associations between cardiometabolic risk and environment measures, using a combination of the criteria of Baron and Kenny [69] and the Monte Carlo Method for Assessing Mediation (MCMAM) [70]. For the food environment, fruit and vegetable intake was entered into models assessing associations between cardiometabolic risk and perceived, objective and overestimated distances to FVR to evaluate (1) associations between diet and each relevant cardiometabolic risk measure (path *b*) accounting for environmental measures; and (2) whether the estimates for the association between environmental and cardiometabolic risk measures were reduced after accounting for fruit and vegetable intake. Associations between access to FVR and diet (path *a*) were also evaluated. A similar process was used to test the mediating role of physical activity on associations between the physical activity environment and cardiometabolic risk. 

Multilevel linear regression was used where diet was the outcome, and multilevel Poisson regression was used where physical activity was the outcome. Where both paths *a* and *b* were statistically significant, mediation was formally tested using the MCMAM approach. This approach estimates the sampling distribution of the mediated (or indirect) effect (*ab*), and corresponding 95% CI in a large number of samples (*n* = 20,000) using a non-parametric bootstrapping procedure. This is a powerful method for assessing mediation where the mediated effect is not normally distributed and the outcome is binary [71]. Mediation was considered significant when the 95% CI did not include zero. 

## 3. Results

A total of 1943 participants responded to the follow-up questionnaire eliciting neighbourhood perceptions (64.9% response rate). Of these, complete perceptions data for FVR and POS and biomedical measures for the cardiometabolic risk outcomes were available for 1773 individuals. Participants missing information on covariates or residential location information, who had changed residential locations (i.e., “moved”) between the Wave 2 clinic visit and the follow-up questionnaire, or were residing outside the greater Adelaide metropolitan region (*n* = 282) were excluded from analyses. The individual- and area-level characteristics of the final number of 1491 participants included for analysis are presented in Table 1. The full (*n* = 1943) and final analytic (*n* = 1491) samples were compared for differences in age, gender, education, income, metabolic syndrome, central obesity, hypertension, dyslipidaemia, prediabetes/diabetes, and distances to the nearest POS and nearest FVR. The total sample (*n* = 1916 with complete data) was slightly younger than the final analytic sample (*n* = 1491; 55.5 (14.9) vs. 56.6 (14.3) years; *p* = 0.03), and those in the total sample (*n* = 1866 with complete data) lived a further distance from their nearest FVR than those included in the final analytic sample (*n* = 1491; 2301.7 (15,867.3) m vs. 1164.6 (881.1) m; *p* = 0.006). The final analytic sample excluded those living outside the Adelaide metropolitan area, and it could be expected that rural residents live a greater distance to resources such as FVR.

The results of regression analyses evaluating associations between the cardiometabolic outcome measures and perceived, objective, and overestimated distances to FVR are presented in Table 2. Greater perceived distances to the nearest FVR were positively associated with odds of having metabolic syndrome and hypertension. Only the association with hypertension, however, remained statistically significant after accounting for objectively measured distance and after adjustment for covariates and fruit and vegetable intake. Overestimating the distance to the nearest FVR was associated with a 36% greater likelihood of having hypertension, after adjustment for covariates and fruit and vegetable intake. Objective FVR access was not associated with any measure of cardiometabolic risk. 

Table 3 presents the associations between perceived, objective, and overestimated distances POS and cardiometabolic outcome measures. The objective distance to POS was inversely associated with odds of having dyslipidaemia, after adjustment for covariates and physical activity, and independent of perceptions of distance to POS. Perceptions of the distance to POS were inversely associated with odds of having hypertension, after adjustment for covariates and physical activity, and independent of the objective access measures. Overestimating the distance to POS was associated with a 42% greater likelihood of having hypertension and a 25% greater likelihood of having dyslipidaemia, after adjusting for covariates and physical activity.

Associations between outcome measures and covariates were as follows (Model 1, Table 2 and Table 3, for all combinations of outcome and predictor measures). Age (*p* < 0.0001–0.04) and gender (*p* < 0.0001–0.03) were associated with all cardiometabolic outcomes for all models, except dyslipidaemia and age where overestimated distances to FVR and POS were the predictors, and prediabetes/diabetes and gender where overestimated FVR distance was the predictor. Education and annual household income were less consistently associated with cardiometabolic outcomes. Education was related to metabolic syndrome (*p* = 0.03), central obesity (*p* = 0.03), and dyslipidaemia (*p* = 0.03) in models accounting for overestimated FVR distance, and perceived and objective POS (central obesity only; *p* = 0.046–0.049). Annual household income was associated with hypertension (*p* = 0.0003–0.04) and dyslipidaemia (*p* = 0.004–0.04) for models, and prediabetes/diabetes where objective distance to FVR (*p* = 0.03) and overestimated POS distance (*p* = 0.04) were the predictors. Area-level income and the number of years lived at one’s current residence were not statistically significant covariates in any multivariable model testing the unique associations between distance measures and cardiometabolic outcomes. 

To test for mediation, relationships between environment measure and behaviour (path *a*), and behaviour and cardiometabolic outcome accounting for the environment measure (path *b*), must both be statistically significant. Neither perceived (*p* = 0.44) nor objective (*p* = 0.26) distances to FVR were related to fruit and vegetable intake. Overestimated distances to FVR were associated with fruit and vegetable intake (*p* = 0.04), but fruit and vegetable intake was not associated with hypertension (*p* = 0.32) accounting for FVR distance measures; thus, criteria for mediation were not met for relationships between FVR and cardiometabolic risk outcomes. 

Perceived (*p* < 0.0001) and overestimated distances to POS (*p* < 0.0001), but not objective distances (*p* = 0.13), were related to physical activity; however, physical activity was not related to either dyslipidaemia (*p* = 0.11) or hypertension (*p* = 0.39), accounting for POS distance measures. Thus, criteria for mediation were not met for POS and cardiometabolic risk associations. 

## 4. Discussion

This study demonstrates associations between objective, perceived and overestimated distances to FVR and POS and specific measures of cardiometabolic risk in an Australian urban-dwelling, population-based sample. Unexpectedly, dyslipidaemia was inversely associated with the objective distance to POS, but positively associated with overestimations of the distance to POS. Hypertension was positively related to perceived and overestimated distances to both FVR and POS. Neither metabolic syndrome itself, nor central obesity or prediabetes/diabetes, were independently related to any objective, perceived or overestimated distance to FVR or POS.

Differences in the relationships between access to resources and cardiometabolic outcomes were observed according to measures of access (perceived, objective or overestimated distances) and outcomes. The differential associations observed here may reflect true differences in the influence of the food and physical activity environment on specific clinical risks. For instance, previous research [72] has demonstrated associations between the food environment and overweight and obesity, but not with diabetes, high cholesterol or hypertension. Similarly, a prospective analysis [16] showed that larger POS and greater walkability were associated with incident prediabetes/diabetes, and that living in areas with an unhealthy food environment index was associated with incident abdominal obesity; however, hypertension and dyslipidaemia were not associated with any food or physical activity environment measures. In contrast with these prior studies, the present study showed associations between food and physical activity environment measures and hypertension and dyslipidaemia, but not with central obesity or prediabetes/diabetes. This could be a result of the different expressions of the food and physical activity environments used (e.g., distance to the nearest POS versus local POS size). These differential relationships between food and physical activity environments and cardiometabolic outcomes could also be a result of the contemporaneous measurement of the environment and health outcomes in this cross-sectional study. For instance, it may take longer to establish a relationship between the environment and metabolic syndrome compared to its individual components, given the requirement for a number of risk factors to be present for a metabolic syndrome diagnosis. The finding that dyslipidaemia was related to low objective distance was unexpected. It could be that this association reflects other, unmeasured environmental factors. Alternatively, this unexpected association may simply be spurious. Future longitudinal research using the same measures of environment and outcomes would assist in understanding whether differential associations between the food and physical activity environment and individual cardiometabolic risk measures are due to longer lag times for some cardiometabolic risk conditions, and whether the associations are truly specific.

The associations between overestimated distances to resources and dyslipidaemia and hypertension are consistent with the results of two previous studies [50,51] demonstrating that discordant, or inaccurate, perceptions of local area resources have implications for cardiometabolic risk. This finding suggests importantly that negative perceptions of access to healthy food and physical activity resources, even where access is objectively assessed as reasonable, are related to cardiometabolic outcomes. It is possible that one’s health, in this case cardiometabolic risk, shapes perceptions, in particular, inaccurate perceptions of the neighbourhood environment. For instance, Gebel and colleagues [73] found that being overweight was associated with misperceiving (i.e., inaccurately perceiving) a high-walkable environment as low-walkable. Reverse causality cannot be ruled out in this cross-sectional study.

Interestingly, the addition of fruit and vegetable consumption and physical activity covariates in statistical models did not greatly alter the estimated associations between distance to FVR and POS and cardiometabolic risk outcomes. Criteria for mediation were not met for any environment-health association. Auchincloss and colleagues [23] found speculative support for a mediating role of physical activity and fruit and vegetable intake in cross-sectional associations between the perceived food and physical activity environment and insulin resistance. However, formal testing of mediation was not undertaken. Two studies that have formally tested the mediating role of physical activity in place–health associations have reported conflicting results, with one study demonstrating a partial mediating role of physical activity between POS size and cardiometabolic risk [74], and another study reporting no mediation by physical activity in associations between perceived convenience of physical activity facilities and weight status [75]. 

The lack of evidence in support of a mediating role for physical activity or fruit and vegetable intake in the present study may be due to two reasons. First, behaviour measures used in the present study may not adequately represent the specific health behaviours of relevance. For instance, access to destinations has been positively associated with an increase in utilitarian walking behaviour over a 12-month period in an Australian prospective analysis [76]. Thus, utilitarian walking may be more likely than walking for recreation or exercise or fruit and vegetable intake to mediate associations between the neighbourhood resource environment and cardiometabolic risk. In addition, work-related physical activity and other activities such as gardening may be relevant physical activity measures. However, data on such alternate forms of physical activity were not collected in from NWAHS participants and could not be accounted for in this study. Second, intermediate mechanisms other than physical activity and fruit and vegetable intake may act to link resources access with cardiometabolic risk. Other potential mechanisms may include unhealthy food intake, or caloric overconsumption. Mechanisms may also include chronic stress resulting from negative perceptions of the local area, specifically, perceptions of living in an under-resourced area. Further inquiry into the mechanisms that explain associations between neighbourhood attributes and health outcomes in longitudinal analyses is required in order to better understand how specific environmental features shape cardiometabolic outcomes.

An important limitation of this study is the cross-sectional nature of the associations evaluated. Prospective analyses are required to evaluate the temporal ordering and direction of the pathways linking objective and perceived environmental attributes to cardiometabolic risk. Additionally, measures of walkability were not included in the present analysis but are conceptually relevant, particularly in relation to perceived distance to resources. Future research is required to explore how objectively assessed walkability influences perceptions of distance to resources. The data available for the perceived built environmental resources for this research limited our ability to determine whether attributes other than distance, for instance count, density or quality of resources, are similarly related to cardiometabolic risk. Future studies should aim to collect resident perceptions of a range of measures of the built and social environment so as to facilitate a better understanding of the role of specific environmental attributes in linking neighbourhoods to health outcomes. Moreover, investigation of further underlying individual and neighbourhood factors that might contribute to explaining the discordance between perceived and objective resource measures, such as physical functioning, mental well-being, or certain features of the neighbourhood context, is essential to better understand relationships between neighbourhood environments, behaviour, and health outcomes, and should be explored in future research.

The main strength of this study was the direct comparison of resident perceptions and objective assessments of the same environmental attributes, namely, distance to the nearest FVR and POS, and their discordance. In addition, the inclusion of analyses investigating inaccurate perceptions of the built environment in relation to clinically measured cardiometabolic risk is a novel contribution to an emerging literature. 

## 5. Conclusions

This study demonstrated limited, specific associations between measures of the food and physical activity environments, particularly perceived and overestimated distances to resources, and clinical cardiometabolic risk outcomes. These findings support the notion that objective and perceived measures of neighbourhood attributes represent distinct environmental constructs and are differently related to clinical cardiometabolic risk outcomes, highlighting the importance of using both perceived and objective measures to provide a richer understanding of how environmental perceptions can shape how objective aspects of environments relate to health. The knowledge generated from this study has implications in particular for an important target for public health intervention: improving perceptions of local access to health-promoting resources. Interventions to improve perceptions of access, such as signposting distances to local facilities and resources along footpaths, in combination with improving access to built environmental resources, have the potential to contribute in an important way to population-level improvement in cardiometabolic risk conditions and subsequent reductions in disease.

## Figures and Tables

**Table 1:** Individual and area characteristics of the sample (*n* = 1491).

Individual Characteristics	Mean (SD)/*n* (%)
**Age** (years)	56.6 (14.3)
**Gender** (*n* (%))	
Male	675 (45.3%)
Female	816 (54.7%)
**Education level (*n* (%))**	
Less than bachelor degree	1298 (87.1%)
Bachelor degree or higher	193 (12.9%)
**Annual household income** **(AUD$) (*n* (%))**	
Less than $20,001	397 (26.6%)
$20,001 to $60,000	700 (47.0%)
More than $60,000	394 (26.4%)
**Duration at current residence** (years)	20.3 (13.9)
**Fruit and vegetable intake** (number of serves per day)	4.2 (1.9)
**Physical activity score (total energy expenditure** (METS))	1709.3 (3119.4)
**Metabolic syndrome** (*n* (%))	552 (37.0%)
**Central obesity** (*n* (%))	1064 (71.4%)
**Hypertension** (*n* (%))	878 (58.9%)
**Dyslipidaemia** (*n* (%))	700 (47.0%)
**Prediabetes/Diabetes** (*n* (%))	729 (48.9%)
**Area Characteristics**	**Mean (SD)/*n* (%)**
**Area-level median weekly household income** (AUD$)	851.38 (200.4)
**Distance to the nearest FVR** (m)	1164.6 (881.1)
**Distance to the nearest POS** (m)	241.9 (300.5)
**Nearest FVR: Perceived distance overestimated objective distance** (*n* (%))	561 (37.6%)
**Nearest FVR: Perceived distance matched objective distance** (*n* (%))	628 (42.1%)
**Nearest POS: Perceived distance overestimated objective distance** (*n* (%))	728 (48.8%)
**Nearest POS: Perceived distance matched objective distance** (*n* (%))	699 (46.9%)

**Table 2:** Associations between cardiometabolic outcomes and perceived, objective, and overestimated distances to fruit and vegetable retailers (FVR).

Cardiometabolic Outcomes	Objective Distance (*n* = 1491)		Perceived Distance (*n* = 1491)		Overestimated Distance (*n* = 1189) ^a^	
	OR (95% CI)	*p*-Value	OR (95% CI)	*p*-Value	OR (95% CI)	*p*-Value
**Metabolic syndrome**						
Model 1	1.08 (0.96, 1.23)	0.197	1.11 (1.01, 1.22)	0.036	1.10 (0.86, 1.42)	0.449
Model 2	1.02 (0.89, 1.18)	0.774	1.10 (0.98, 1.22)	0.093	-	-
Model 3	1.03 (0.89, 1.18)	0.713	1.10 (0.98, 1.22)	0.103	1.08 (0.84, 1.40)	0.539
**Central obesity**						
Model 1	1.11 (0.97, 1.26)	0.118	1.09 (0.98, 1.20)	0.098	1.22 (0.93, 1.59)	0.148
Model 2	1.07 (0.92, 1.24)	0.407	1.06 (0.94, 1.19)	0.326	-	-
Model 3	1.07 (0.92, 1.24)	0.386	1.06 (0.94, 1.19)	0.342	1.21 (0.93, 1.59)	0.157
**Hypertension**						
Model 1	0.98 (0.86, 1.12)	0.763	1.13 (1.02, 1.25)	0.022	1.37 (1.03, 1.82)	0.029
Model 2	0.87 (0.75, 1.02)	0.089	1.19 (1.05, 1.34)	0.005	-	-
Model 3	0.88 (0.75, 1.02)	0.099	1.19 (1.05, 1.34)	0.005	1.36 (1.02, 1.80)	0.034
**Dyslipidaemia**						
Model 1	1.08 (0.96, 1.22)	0.176	1.08 (0.98, 1.18)	0.108	1.14 (0.89, 1.45)	0.300
Model 2	1.04 (0.91, 1.19)	0.532	1.06 (0.95, 1.17)	0.284	-	-
Model 3	1.05 (0.92, 1.20)	0.501	1.06 (0.95, 1.17)	0.298	1.13 (0.88, 1.44)	0.342
**Prediabetes/Diabetes**						
Model 1	1.03 (0.91, 1.16)	0.658	0.99 (0.90, 1.09)	0.868	0.82 (0.63, 1.07)	0.139
Model 2	1.05 (0.91, 1.21)	0.543	0.97 (0.87, 1.09)	0.653	-	-
Model 3	1.05 (0.91, 1.21)	0.515	0.97 (0.87, 1.09)	0.635	0.82 (0.63, 1.06)	0.126

OR: odds ratio; 95% CI: 95% confidence interval. Model 1 adjusted for participant age, gender, household income, educational attainment, duration at current residence, and area-level income. Model 2 additionally included both objective and perceived distance to the nearest FVR. Model 3 additionally adjusted for fruit and vegetable intake. ^a^ Sample for associations between overestimated distances and cardiometabolic risk factors excludes *n* = 302 participants that underestimated distances.

**Table 3:** Associations between cardiometabolic outcomes and perceived, objective, and overestimated distances to public open spaces (POS).

Cardiometabolic Outcomes	Objective Distance (*n* = 1491)		Perceived Distance (*n* = 1491)		Overestimated Distance (*n* = 1427)	
	OR (95% CI)	*p*-Value	OR (95% CI)	*p*-Value	OR (95% CI)	*p*-Value
**Metabolic syndrome**						
Model 1	0.92 (0.73, 1.16)	0.463	1.08 (0.99, 1.18)	0.097	1.22 (0.97, 1.55)	0.120
Model 2	0.87 (0.69, 1.10)	0.251	1.09 (1.00, 1.20)	0.060	-	-
Model 3	0.87 (0.69, 1.10)	0.248	1.09 (0.99, 1.20)	0.067	1.21 (0.96, 1.53)	0.114
**Central obesity**						
Model 1	0.99 (0.79, 1.25)	0.953	1.04 (0.94, 1.15)	0.478	1.13 (0.88, 1.44)	0.337
Model 2	0.97 (0.76, 1.23)	0.794	1.04 (0.94, 1.16)	0.451	-	-
Model 3	0.97 (0.76, 1.23)	0.807	1.04 (0.94, 1.15)	0.475	1.12 (0.87, 1.43)	0.369
**Hypertension**						
Model 1	0.91 (0.72, 1.16)	0.453	1.12 (1.01, 1.25)	0.036	1.43 (1.12, 1.84)	0.005
Model 2	0.84 (0.66, 1.07)	0.161	1.15 (1.03, 1.28)	0.016	-	-
Model 3	0.84 (0.66, 1.07)	0.166	1.14 (1.02, 1.28)	0.018	1.42 (1.11, 1.83)	0.006
**Dyslipidaemia**						
Model 1	0.80 (0.65, 1.00)	0.051	1.05 (0.96, 1.15)	0.292	1.26 (1.01, 1.58)	0.039
Model 2	0.77 (0.62, 0.96)	0.023	1.08 (0.98, 1.18)	0.112	-	-
Model 3	0.77 (0.62, 0.96)	0.023	1.07 (0.98, 1.18)	0.123	1.25 (1.00, 1.57)	0.048
**Prediabetes/Diabetes**						
Model 1	1.07 (0.85, 1.34)	0.566	0.99 (0.90, 1.09)	0.907	0.99 (0.78, 1.26)	0.942
Model 2	1.08 (0.85, 1.36)	0.530	0.99 (0.89, 1.09)	0.780	-	-
Model 3	1.08 (0.85, 1.36)	0.525	0.99 (0.89, 1.09)	0.766	0.99 (0.78, 1.25)	0.922

OR: odds ratio; 95% CI: 95% confidence interval. Model 1 adjusted for participant age, gender, household income, educational attainment, duration lived at current residence, and area-level income. Model 2 additionally included both objective and perceived distance to the nearest POS. Model 3 additionally adjusted for physical activity.
